# Identification of risk factors for postoperative stage 3 acute kidney injury in patients who received surgical repair for acute type A aortic dissection

**DOI:** 10.1186/s12893-022-01526-x

**Published:** 2022-03-02

**Authors:** Zhigang Wang, Min Ge, Zheyun Wang, Cheng Chen, Lichong Lu, Lifang Zhang, Dongjin Wang

**Affiliations:** 1 Department of Cardio-thoracic Surgery, Affiliated Drum Tower Hospital, Medical School of Nanjing University, Zhongshan Road 321, Nanjing, 210008 China; 2 Department of Psychiatry, The First Affiliated Hospital, Zhengzhou University, Zhengzhou, China

**Keywords:** Acute kidney injury, Type A aortic dissection, Outcomes

## Abstract

**Background:**

Acute kidney injury (AKI) is a serious complication that often occurred after acute type A aortic dissection (ATAAD) surgery. Previous studies proved that the Kidney Disease Improving Global Outcomes (KDIGO) defined stage 3 AKI was associated with lower long-term survival rate. However, the risk factors for developing stage 3 AKI had not been identified. The aim of the study was to explore the risk factors for developing KDIGO stage 3 after ATAAD operation.

**Methods:**

This study included 993 patients who received ATAAD operation from 2014 to 2019 at the Nanjing Drum Tower Hospital. Postoperative AKI was diagnosed according to the KDIGO criteria. Multivariate logistic regression analyses were applied to identify risk factors for stage 3 AKI. Kaplan–Meier survival analyses and Cox proportional hazards regression model were conducted to explore the association between different AKI stages and postoperative survival rate.

**Results:**

The mean age of all enrolled patients was 53.0 ± 13.1 years. A total of 236 (23.8%) patients suffered postoperative stage 3 AKI including 165 patients who required renal replacement therapy. Advanced age (odds ratio [OR] 1.031; 95% confidence interval [CI] 1.005–1.057; *P* = 0.018), prolonged cardiopulmonary bypass (CPB) duration (OR 1.010; 95% CI 1.002–1.018; *P* = 0.013), and higher drainage volume 24 h after surgery (OR 1.000; 95% CI 1.000–1.001; *P* = 0.033) were identified as independent risk factors for developing stage 3 AKI. In addition, our result showed that the mortality rate was correlated significantly with the severity of AKI defined by KDIGO criteria and the Cox regression analysis showed that only stage 3 AKI, but not stage 1 and 2, was an independent risk factor for mortality (Hazard ratio 10.365, 95% CI 4.208 to 25.528; *P* < 0.001) after adjusting for important confounding factors.

**Conclusions:**

Our study suggested that stage 3 postoperative AKI was significantly associated with decreased postoperative survival rate after ATAAD surgery. Advanced age, increased CPB duration and drainage volume 24 h after surgery were identified as risk factors for developing stage 3 AKI.

## Introduction

It is well accepted in the field that acute kidney injury (AKI) is a common and serious complication after acute type A aortic dissection (ATAAD) surgery. However, the incidence of AKI after aortic arch surgery varied widely (20–67%) in previous studies largely due to the lack of consensus in AKI definition until 2012 when the Kidney Disease Improving Global Outcome (KDIGO) criteria was published [1–5].

Inconsistent association between the development of postoperative AKI and long-term adverse outcomes in patients with ATAAD had been suggested in previous studies. Some studies demonstrated that only stage 3 AKI was significantly associated with lower long-term survival [6, 7], whereas others have found all stages postoperative AKI increased the mortality of patients with ATAAD [2, 3, 8]. However, most of these studies were conducted in a relatively small population and designed differently.

The current study not only aimed to decipher the association between different stages of AKI and disease outcomes but also to identify the risk factors for developing stage 3 AKI in patients after receiving ATAAD surgery. We present the following article in accordance with the STROBE reporting checklist.

## Materials and methods

### Study population

We retrospectively reviewed the medical records of 1052 patients who received ATAAD surgery at Nanjing Drum Tower Hospital between January 2014 and December 2019. Patients who were admitted to our hospital more than 48 h after symptom onset were excluded from the analysis (n = 13). Patients who required renal replacement therapy (RRT) before surgery (n = 27) were also excluded due to difficulty in measuring the progression of renal dysfunction. Patients who died during surgery or within 24 h after surgery (n = 19) were excluded when the death was considered irrelevant with postoperative renal dysfunction. The protocol of this study was approved by the institutional review board and individual consent was waived considering the retrospective nature of the study.

The stage 3 AKI was defined according to the serum creatinine (sCr) component of the KDIGO criteria at day 7 after the surgery (> 3.0 × baseline sCr or increase in sCr to ≥ 4.0 mg/dL or initiation of RRT) (Table 1). This study did not apply the urine output as the criteria in defining stage 3 AKI considering the high risk of inaccuracy in this retrospective study. Preoperative hepatic dysfunction was diagnosed in patients with Model of End-Stage Liver Disease (MELD) score ≥ 12. Routine follow up of patients’ general health status was conducted by telephone contact since 2014 once every year. If patients had died at the time of contact, the date and the cause of deaths was collected. All patients were divided into the stage 3 AKI group and the control group.

**Table 1:** Kidney Disease Improving Global Outcomes (KDIGO) criteria for acute kidney injury

Stage	Serum creatinine (sCr) increase
1	1.5–1.9 times baseline or ≥ 0.3 mg/dL increase
2	2.0–2.9 times baseline
3	> 3.0 times baseline or increase in sCr to ≥ 4.0 mg/dL or initiation of renal replacement therapy

### Surgical procedure

The surgical techniques applied in the study was described in detail in a previous study [9]. Briefly, the root procedures included direct repair or replacement of inclusion root, Bentall procedure, or David procedure. The distal arch repair consisted of hemi-arch replacement, island arch replacement, total arch replacement, triple-branched stent, and fenestrated stent depending on patient’s preoperative status, entry tear location, and aortic diameter, as described previously.

### Statistical analysis

Continuous variables were expressed as mean ± standard deviation or median with interquartile range as appropriate. Categorical variables were presented as frequencies with percentages. The χ^2^ test or Fisher exact test was used for comparing categorical variables, whereas the *t*-test or Mann–Whitney *U*-test was chose for examining continuous variables.

To reduce selection bias, one-to-one propensity score (PS) matching was conducted between two groups. All the pre-operative variables listed in Table 2 and intro-operative variables listed in Table 3 were included in the analysis. The PS for each patient was estimated using the logistic regression model and matched to the nearest neighboring point with a tolerance level on the maximum PS distance (callipers of width 0.2 standard deviations of the logit of the PS).

**Table 2:** Baseline characteristics of the unmatched and propensity matched groups

Variables	Total (n = 993)	Overall cohort			PSM Cohort		
		Stage 3 (n = 236)	Control (n = 757)	*P* value*	Stage 3 (n = 119)	Control (n = 119)	*P* value*
Demographic data							
Age (year)	53.0 ± 13.1	54.0 ± 13.4	52.7 ± 13.1	0.186	53.7 ± 13.3	51.2 ± 12.3	0.141
Male (%)	730 (73.5)	179 (75.8)	551 (72.8)	0.352	92 (77.3)	93 (78.2)	0.876
BMI (kg/m^2^)	25.6 ± 4.8	26.3 ± 4.8	25.4 ± 4.8	0.036	26.5 ± 4.8	26.0 ± 4.4	0.371
Medical history							
Hypertension (%)	715 (72.0)	186 (78.8)	529 (69.9)	0.008	87 (73.1)	91 (76.5)	0.550
Diabetes mellitus (%)	23 (2.3)	4 (1.7)	19 (2.5)	0.467	0 (0)	3 (2.5)	0.247
Previous cardiac surgery (%)	44 (4.4)	14 (5.9)	30 (4.0)	0.199	6 (5.0)	5 (4.2)	0.758
Previous coronary artery disease (%)	34 (3.4)	11 (4.7)	23 (3.0)	0.231	3 (2.5)	2 (1.7)	1.000
Cerebrovascular disease (%)	36 (3.6)	9 (3.8)	27 (3.6)	0.859	6 (5.0)	1 (0.8)	0.119
LVEF (%)	55.1 ± 6.6	54.2 ± 5.9	55.3 ± 6.7	0.489	55.1 ± 5.6	54.0 ± 8.7	0.728
Time from symptom onset to admission	10.7 ± 6.0	12.3 ± 7.1	11.4 ± 6.4	0.274	12.5 ± 6.8	11.7 ± 6.5	0.259
Pericardial tamponade (%)	166 (16.7)	55 (23.3)	111 (14.7)	0.002	21 (17.6)	17 (14.3)	0.479
DeBakey type I (%)	816 (82.2)	207 (87.7)	609 (80.4)	0.011	103 (86.6)	102 (85.7)	0.851
MELD score ≥ 12 (%)	268 (27.2)	103 (45.2)	165 (21.8)	< 0.001	38 (33.0)	31 (26.1)	0.241
Preoperative laboratory data							
WBC (10^9^/L)	11.7 ± 11.1	12.3 ± 4.7	11.5 ± 12.4	0.339	12.9 ± 5.2	11.6 ± 4.2	0.171
Hemoglobin (g/L)	122.4 ± 29.0	121.4 ± 41.2	122.8 ± 24.0	0.630	120.8 ± 48.7	117.4 ± 26.6	0.381
PLT (10^9^/L)	150.9 ± 86.2	142.6 ± 62.7	153.5 ± 92.3	0.091	151.8 ± 59.0	145.7 ± 62.4	0.440
sCr (μmol/L)	81.0 (61.8, 114.2)	101.2 (68.1, 175.8)	77.6 (60.0, 104.1)	< 0.001	111.1 ± 84.4	108.0 ± 88.3	0.784
Bun (mmol/L)	8.2 ± 4.0	9.9 ± 5.6	7.7 ± 3.2	< 0.001	8.3 ± 3.9	8.6 ± 4.1	0.658
ALB (g/L)	36.5 ± 5.0	35.6 ± 5.9	36.8 ± 4.7	0.008	36.5 ± 5.8	36.3 ± 4.9	0.758
ALT (U/L)	25.6 (15.7, 46.8)	30.3 (15.2, 81.1)	22.3 (14.3, 41.1)	< 0.001	29.6 (17.4, 74.9)	25.9 (14.2, 41.5)	0.113
CRP (mg/dL)	23.6 (5.0, 81.6)	19.2 (4.8, 66.4)	22.2 (4.4, 67.5)	0.628	19.2 (5.2, 67.2)	24.9 (4.5, 106.4)	0.171
Triglyceride (mmol/L)	1.0 (0.7, 1.5)	1.1 (0.7, 1.6)	0.9 (0.7, 1.5)	0.166	1.1 (0.8, 1.6)	1.0 (0.8, 1.4)	0.353
Total bilirubin (μmol/L)	15.8 (11.1, 23.1)	14.6 (10.0, 23.1)	15.2 (11.3, 22.2)	0.685	17.7 ± 15.4	18.5 ± 15.4	0.674
INR	1.1 (1.0, 1.2)	1.3 ± 0.8	1.2 ± 0.7	< 0.001	1.2 ± 0.5	1.1 ± 0.2	0.060
D-dimer (ng/mL)	4.6 (2.3, 9.3)	4.9 (2.9, 12.3)	3.9 (2.1, 7.1)	0.001	4.7 (2.9, 9.5)	6.0 (3.1, 11.5)	0.868
Preoperative CTA							
Involving the left renal artery (%)	394 (77.0)	91 (77.0)	303 (79.7)	0.419	45 (73.8)	44 (72.1)	0.596
Involving the right renal artery (%)	440 (87.6)	100 (85.4)	340 (89.9)	0.386	53 (78.5)	45 (74.2)	0.479

Data presented as n (%), median (IQR), or mean ± standard deviation

*BMI* body mass index; *LVEF* left ventricular ejection fraction; *MELD* model of end-stage liver disease; *WBC* white blood cell; *PLT* platelet; *sCr* serum creatinine; *BUN* blood urea nitrogen; *ALB* albumin; *ALT* alanine transaminase; *CRP* c-reactive protein; *INR* international normalized ratio; *CTA* computed tomography angiography; *PSM* propensity score matching

*P values indicate differences between stage 3 AKI and control group patients. *P* < 0.05 was considered statistically significant

**Table 3:** Operative variables of the unmatched and propensity matched groups

Variables	Total (n = 993)	Overall cohort			PSM Cohort		
		Stage 3 (n = 236)	Control (n = 757)	*P* value*	Stage 3 (n = 119)	Control (n = 119)	*P* value*
TAR (%)	478 (48.1)	131 (55.5)	347 (45.8)	0.009	71 (59.7)	75 (63.0)	0.594
CABG (%)	59 (5.9)	22 (9.3)	37 (4.9)	0.012	12 (10.1)	10 (8.4)	0.654
CPB time (min)	233.8 ± 68.0	258.6 ± 74.6	226.1 ± 63.9	< 0.001	258.3 ± 66.2	261.8 ± 73.3	0.701
Aortic cross-clamp time (min)	164.2 ± 55.6	176.7 ± 61.6	160.3 ± 53.1	< 0.001	180.8 ± 62.2	182.9 ± 61.7	0.801
DHCA time (min)	29.3 ± 12.9	32.1 ± 13.5	28.5 ± 12.6	< 0.001	32.0 ± 14.5	29.8 ± 11.8	0.216

Data presented as n (%), median (IQR), or mean ± standard deviation

*TAR* total arch replacement; *CABG* coronary artery bypass graft; *CPB* cardiopulmonary bypass; *DHCA* deep hypothermic circulatory arrest; *PSM* propensity score matching

*P values indicate differences between stage 3 AKI and control group patients. *P* < 0.05 was considered statistically significant

Multivariate logistic regression analysis was performed (stepwise enter method) using variables identified in the univariable analyses (*P*-value < 0.20) to identify independent risk factors for developing stage 3 AKI after surgery. We used Kaplan–Meier methods and multivariate Cox proportional hazards regression to assess the impact of AKI for postoperative mortality. For Cox regression analysis, all variables with a *P*-value < 0.20 identified in univariable analysis were included in the model. A forward stepwise procedure was applied to introduce variables to the final models. A *P*-value less than 0.05 was considered statistically significant. All analyses were conducted with SPSS software (version 25, SPSS Inc, Chicago, IL).

## Results

A total of 993 patients were eventually enrolled in this study. The patients were predominantly male (73.5%), with a mean age of 53 ± 13 years (range 19–87). Postoperative AKI was identified in 528 patients (53.6%) including 161 (16.2%) stage 1, 131 (13.2%) stage 2, and 236 (23.8%) stage 3. A total of 238 patients (119 pairs) selected by this PS-based matching procedure with similar baseline characteristics were eventually enrolled in the analysis.

Patient baseline demographic information was presented in Table 2. For the unmatched patients, compared to the control group, patients with increased body weight and hypertension diagnosis were more commonly occurred in the stage 3 AKI group. Unsurprisingly, the baseline serum creatinine (sCr) level was significantly increased among patients in the stage 3 AKI group compared to the control group (*P* < 0.001). However, no significant difference of time from symptom onset to admission and renal artery involvement was observed between two groups. In the PS model, the baseline characteristics of the PS matched groups did not reveal any significant differences.

Patient surgical parameters were shown in Table 3. Before PS matching, our data indicated that the cardiopulmonary bypass (CPB) time (*P* < 0.001), aortic cross-clamp time (*P* < 0.001), and deep hypothermic circulatory arrest time (*P* < 0.001) were all significantly prolonged in patients who developed stage 3 AKI. After PS matching, the intro-operative variables showed no significant differences between the two groups.

The postoperative outcomes are summarized in Table 4. Before matching, a total of 165 patients (16.6%) required temporary RRT after surgery and 86 (52.1%) of them developed dialysis-dependent end-stage renal disease. Furthermore, mechanical ventilation duration, intensive care unit (ICU), and hospital stay were prolonged in patients who developed stage 3 AKI compared to controls. Furthermore, the 30-Day mortality was 30.5% in the stage 3 group and 7.5% in the control group with a significant difference. In the PS matched analysis, ICU stay time and the 30-Day mortality consistently showed significant difference between two groups.

**Table 4:** Postoperative variables of the unmatched and propensity matched groups

Variables	Total (n = 993)	Overall cohort			PSM cohort		
		Stage 3 (n = 236)	Control (n = 757)	*P* value*	Stage 3 (n = 119)	Control (n = 119)	*P* value*
Drainage volume 24 h after surgery (mL)	720.5 ± 650.0	950.0 ± 839.1	652.3 ± 565.2	< 0.001	975.0 ± 926.4	790.4 ± 729.1	0.099
Re-exploration for bleeding (%)	36 (3.6)	15 (6.4)	21 (2.8)	0.010	8 (6.7)	2 (1.7)	0.053
Dialysis (%)	165 (16.6)	165 (69.9)	0 (0)	< 0.001	80 (67.2)	0 (0)	< 0.001
Ventilation time (hour)	17.0 (11.0, 43.0)	26.0 (15.0, 90.0)	15.0 (11.0, 28.8)	< 0.001	34.0 (16.2, 106.8)	24.0 (14.2, 62.0)	0.067
Stroke (%)	85 (8.6)	24 (10.2)	61 (8.1)	0.311	15 (12.6)	12 (10.1)	0.540
Paraplegia (%)	32 (3.2)	20 (8.5)	12 (1.6)	< 0.001	8 (6.7)	3 (2.5)	0.123
Tracheostomy (%)	45 (4.5)	23 (9.7)	22 (2.9)	< 0.001	9 (7.6)	7 (5.9)	0.605
Deep sternal wound infection (%)	14 (1.4)	7 (3.0)	7 (0.9)	0.029	5 (4.2)	1 (0.8)	0.213
30-day mortality (%)	115 (11.6)	72 (30.5)	43 (5.7)	< 0.001	39 (32.8)	22 (18.5)	0.012
ICU Stay time (day)	5.0 (3.0, 8.0)	6.0 (3.5, 11.5)	4.0 (3.0, 5.0)	< 0.001	9.4 ± 8.3	5.8 ± 5.1	< 0.001
Hospital stay time (day)	21.4 ± 12.3	24.3 ± 16.3	20.5 ± 10.7	0.001	24.0 ± 16.7	22.1 ± 14.8	0.351

Data presented as n (%), median (IQR), or mean ± standard deviation

*ICU* intensive care unit; *PSM* propensity score matching

*P values indicate differences between stage 3 AKI and control group patients. *P* < 0.05 was considered statistically significant

Next, multivariate logistic analyses were conducted which suggested that advanced age (odds ratio [OR] 1.031; 95% confidence interval [CI] 1.005–1.057; *P* = 0.018), prolonged CPB duration (OR 1.010; 95% CI 1.002–1.018; *P* = 0.013), and higher drainage volume 24 h after surgery (OR 1.000; 95% CI 1.000–1.001; *P* = 0.033) were independent risk factors for developing postoperative stage 3 AKI (Table 5).

**Table 5:** Multivariable analysis of risk factors for stage 3 acute kidney injury with KDIGO

Variable	OR	95% CI	*P* value
Age (year)	1.031	1.005–1.057	0.018
BMI (kg/m^2^)	1.063	0.993–1.138	0.079
Hypertension	1.033	0.539–1.982	0.921
Previous cardiac surgery	1.110	0.309–3.986	0.837
Pericardial tamponade	1.268	0.451–3.567	0.653
MELD score ≥ 12	1.883	0.758–4.679	0.173
PLT (10^9^/L)	1.002	0.998–1.007	0.338
sCr (μmol/L)	1.005	1.000–1.010	0.053
Bun (mmol/L)	0.958	0.863–1.063	0.417
ALB (g/L)	0.946	0.885–1.012	0.104
Triglyceride (mmol/L)	1.238	0.985–1.557	0.067
INR	1.689	0.698–4.084	0.245
Total bilirubin (μmol/L)	0.993	0.972–1.015	0.518
D-dimer (ng/mL)	1.009	0.993–1.024	0.266
TAR	1.695	0.868–3.308	0.122
CABG	1.850	0.540–6.336	0.327
CPB time (min)	1.010	1.002–1.018	0.013
Aortic cross-clamp time (min)	0.996	0.988–1.005	0.419
DHCA time (min)	1.019	0.992–1.046	0.163
Drainage volume 24 h after surgery (mL)	1.000	1.000–1.001	0.033
Paraplegia	2.578	0.583–11.390	0.212

*P* < 0.05 was considered statistically significant

*BMI*, body mass index; *MELD* model of end-stage liver disease; *PLT* platelet; *SCr* serum creatinine; *ALB* albumin; *INR* international normalized ratio; *TAR* total arch replacement; *CABG* coronary artery bypass graft; *CPB* cardiopulmonary bypass; *DHCA* deep hypothermic circulatory arrest; *OR* odds ratio; *CI* confidence interval

**Fig. 1:**
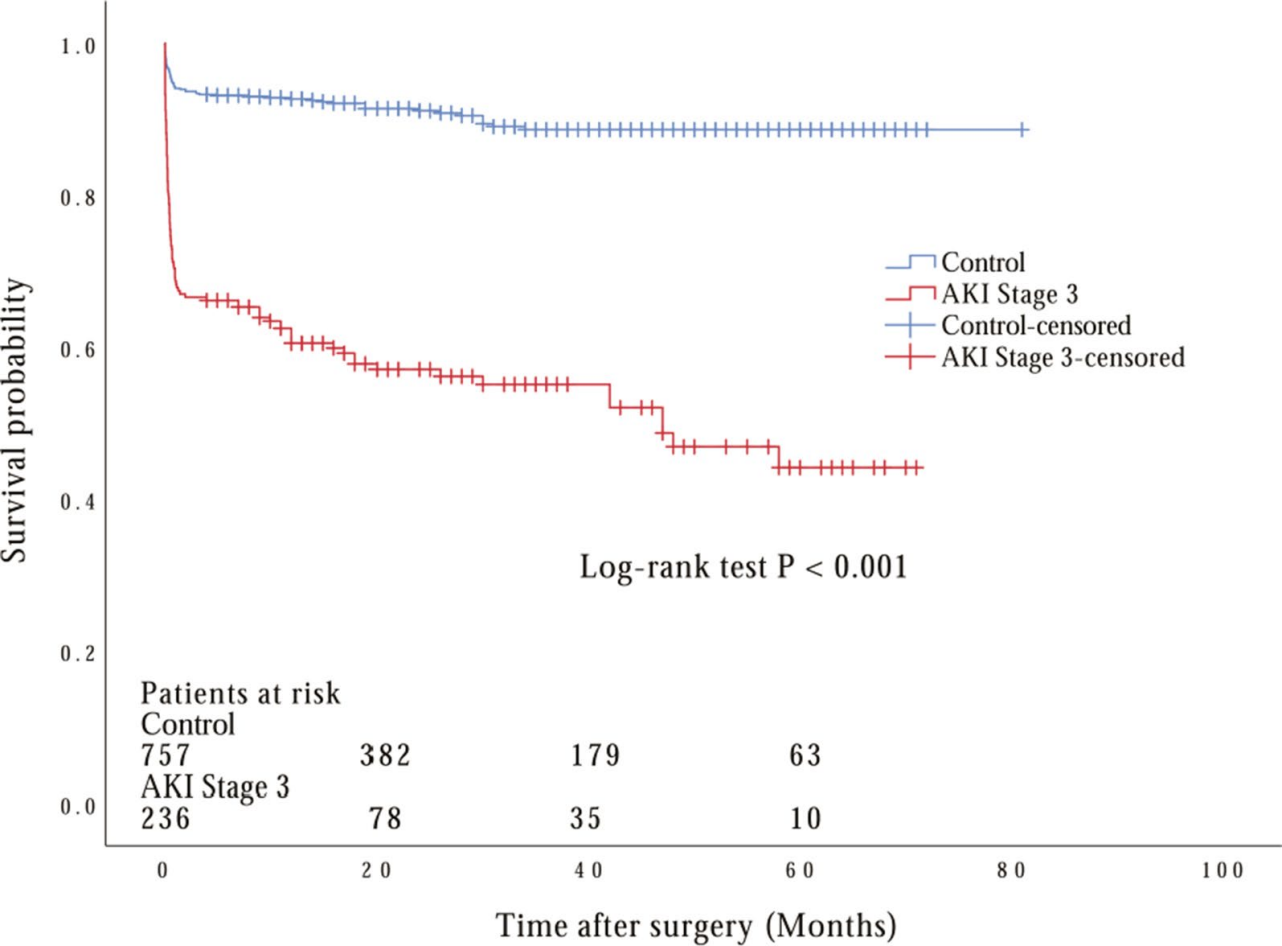
Long-term survival estimates with the use of Kaplan–Meier method after operation for acute type A aortic dissection by severity of acute kidney injury (AKI). Kaplan–Meier estimation before propensity score matching. Significant overall difference is observed (*P* < 0.001 by log-rank test)

**Fig. 2:**
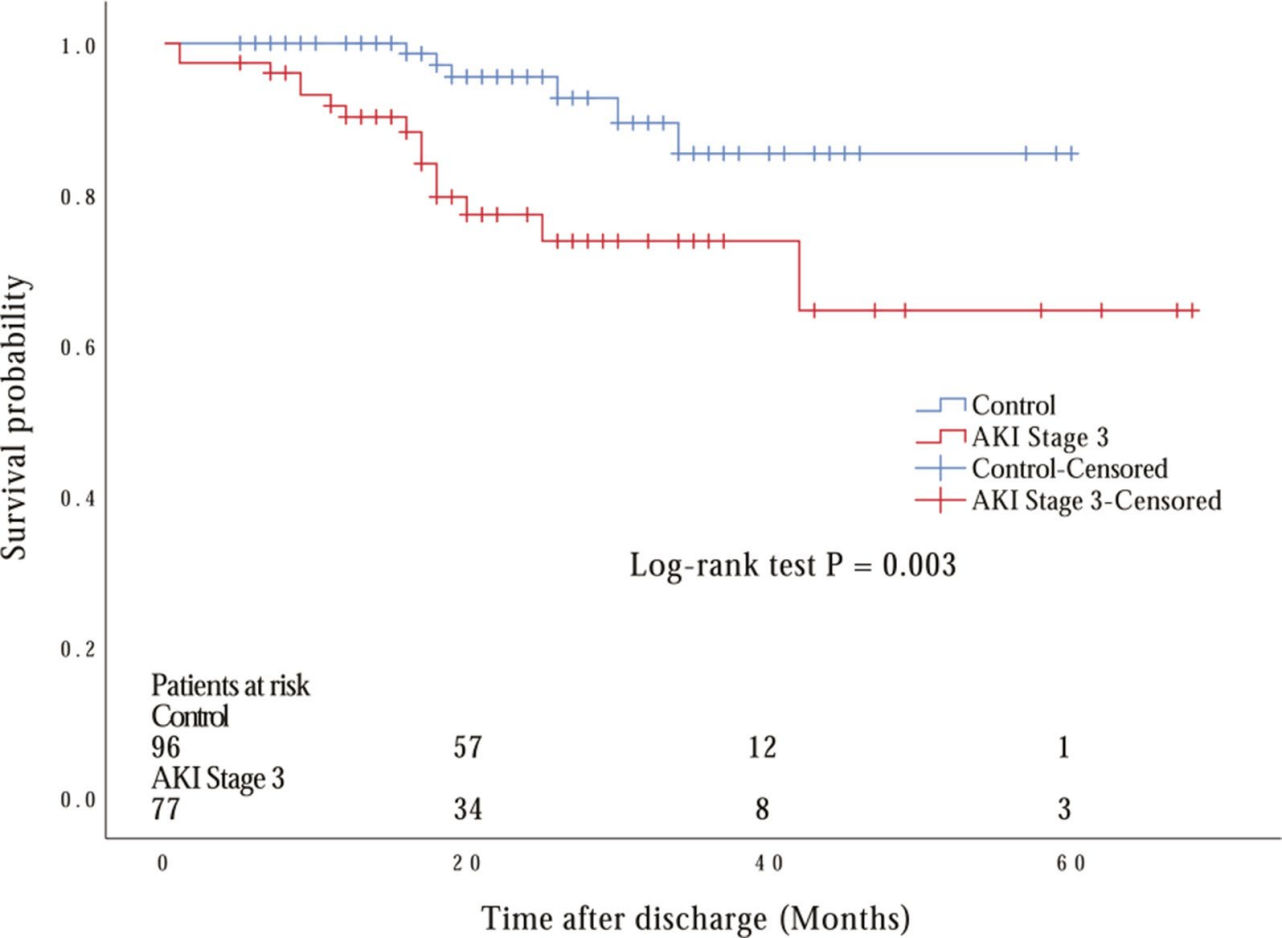
Long-term survival estimates with the use of Kaplan–Meier method after discharge from hospital for acute type A aortic dissection by severity of acute kidney injury (AKI). Kaplan–Meier estimation after propensity score matching. Significant overall difference is observed (*P* = 0.003 by log-rank test)

The follow-up of long-term mortality started on the day of surgery. 76 patients in the stage 3 AKI group and 45 patients in the control group died during the hospitalization period. As a result, a total of 872 patients survived the early postoperative period. The median follow-up time of this cohort was 20 months during which time a total of 11 patients (1.3%) who did not develop postoperative AKI, 8 (0.9%) with stage 1 AKI, 5 (0.6%) with stage 2 AKI, and 29 (3.3%) with stage 3 AKI passed away. 53 patients (6.1%) who were lost to follow-up and 1 patient who committed suicide after hospital discharge were identified as censored data in the following outcome analysis. Our data indicated that the mortality correlated significantly with the severity of AKI defined by KDIGO criteria before and after matching (*P* < 0.001, *P* = 0.003; by log-rank test respectively), which were shown in Figs. 1 and 2. Multivariate Cox analysis for mortality (Table 6) revealed that the only stage 3 AKI (Hazard ratio 10.365; 95% CI 4.208–25.528; *P* < 0.001), but not stage 1 and 2, was a significant and independent risk factor after adjusting for other major clinical factors that might increase postoperative AKI rate.

**Table 6:** Multivariate Cox analysis for mortality

	Hazard ratio	95% CI	*P* value
Age (year)	1.021	0.997–1.046	0.085
Hypertension	1.592	0.728–3.484	0.244
Preoperative hemoglobin (g/L)	0.993	0.982–1.005	0.240
Preoperative CRP (mg/dL)	0.998	0.992–1.004	0.454
Preoperative sCr (μmol/L)	0.998	0.995–1.001	0.254
Preoperative D-dimer (ng/mL)	0.990	0.967–1.014	0.419
Pericardial tamponade	2.280	0.749–6.944	0.147
CPB time (min)	1.003	0.998–1.008	0.241
KDIGO stage 1	2.260	0.710–7.196	0.167
KDIGO stage 2	2.409	0.747–7.765	0.141
KDIGO stage 3	10.365	4.208–25.528	< 0.001
Ventilation time (hour)	1.002	0.998–1.007	0.307

*P* < 0.05 was considered statistically significant

*CRP* c-reactive protein; *sCr* serum creatinine; *CPB* cardiopulmonary bypass; *KDIGO* Kidney Disease Improving Global Outcomes; *CI* confidence interval

## Discussion

In this study, we explored the consequences and risk factors for developing stage 3 AKI after ATAAD surgery. Compared with previous studies, our cohort was much larger and we applied the most updated and well accepted KDIGO criteria to define AKI. We found that advanced age, prolonged CPB duration, and higher drainage volume 24 h after surgery were independent predictors for developing postoperative stage 3 AKI. In addition, consistent with previous studies [6, 7], our study confirmed that only stage 3 AKI, but not stage 1 or 2, was associated with a higher postoperative mortality.

This correlation between AKI severity and long-term survival was also observed in other cardiothoracic operations [5]. A previous meta-analysis discovered that patients who suffered postoperative AKI were more likely to develop chronic kidney disease and end-stage renal disease, and the risk increased with the severity of AKI [10]. Therefore, above results underline the importance of identifying AKI in its early stages and offering appropriate treatment promptly before disease progression.

The incidence of postoperative AKI varied widely among different studies, partially due to the use of different AKI diagnostic criteria. A recent study reported that 24.0% of all patients developed stage 3 AKI after total arch replacement surgery with deep hypothermic circulatory arrest, including 15.4% patients who were treated with RRT [11], which were consistent with our results. However, Ko et al. reported that the incidence of developing stage 3 AKI after aortic arch surgery was 14% and only 9% required RRT [7]. The lower rates might partially due to that patients who received emergency surgery were excluded from that study [12].

Consistent with previous studies, we identified that increased age was an independent risk factor for developing postoperative stage 3 AKI [2, 3, 13]. It has been well accepted in the field that advanced age is associated with structural and functional changes in the kidney including parenchymal mass loss, progressive glomerulosclerosis, tubulopathy, interstitial fibrosis, and afferent-efferent arteriolar shunts, which could all result in reduced renal function [14]. The reduced renal function rendered these patients more vulnerable to the hemodynamic changes during the surgery and it was not surprising that patients with advanced age were more likely to develop postoperative AKI after ATAAD surgery.

CPB duration was another well accepted independent risk factor for postoperative AKI in emergent thoracic aortic surgery. Englberger et al. discovered that longer CPB time (per 10 min) was associated with increasing postoperative AKI occurrence after studying 851 patients who received elective thoracic aortic operation [15]. Similarly, Roh et al. found that longer CPB duration was an independent risk factor for postoperative AKI among patients who received graft replacement of the thoracic aorta [3]. Furthermore, this correlation has been confirmed in some other studies [16, 17] and might be explained at least partially by the non-pulsatile blood flow and activation of inflammatory factors during the bypass [18]. However, more mechanism studies are needed to better understand this association before modification of the surgery process can be made.

Drainage volume 24 h after surgery was identified as another independent risk factor for postoperative stage 3 AKI by the logistic regression model in this study, which had been already recognized as a risk factor for AKI in previous studies [19]. Increases drainage volume can disrupt the homeostasis and induce pro-inflammatory states as well as increase oxidative stress which both can contribute to the pathogenesis of AKI [20]. Increasing evidences suggested that major perioperative transfusions of large amounts of packed red blood cells units and platelet units were associated with an increased incidence of AKI [7, 21, 22]. In line with this study, a recently published study showed that preoperative dual antiplatelet therapy increased the risk of major bleeding and transfusions, which ultimately increased the risk of AKI [23]. Therefore, decreasing 24 h drainage volume is important and may reduce the incidence of postoperative AKI.

## Limitations

This study has some limitations. Firstly, although the sample size was large, the cohort was not homogeneous and might include some confounding factors in the baseline that were not recognized in the study. Secondly, our surgical technique evolved over the long study period, and surgeons with different surgical skills and experiences might also influence our results. Thirdly, the urine output was not examined in this study therefore the number of postoperative AKI patients might be underestimated. Therefore, further prospective multicenter studies are needed to better understand the association between postoperative AKI and disease prognosis before more effective strategies can be recommended.

## Conclusions

This large cohort study underscores the high incidence of postoperative stage 3 AKI following ATAAD repair surgery and its association with worse outcome. Advanced age, prolonged CPB time and increasing drainage volume 24 h after surgery were independent risk factors for developing postoperative stage 3 AKI.

## Data Availability

Data sharing in the current study are available from the corresponding author on reasonable request.

## Abbreviations

AKI: Acute kidney injury
ATAAD: Acute type A aortic dissection
KDIGO: Kidney Disease Improving Global Outcomes
OR: Odds ratio
CI: Confidence interval
CPB: Cardiopulmonary bypass
SCr: Serum creatinine
